# Pulmonary diffuse large B-cell lymphoma with concurrent organizing pneumonia: a case report

**DOI:** 10.3389/fmed.2025.1599268

**Published:** 2025-08-06

**Authors:** Xiaofeng Lu, Dekun Zhou, Li Zou, Guoqi Zhou

**Affiliations:** Department of Pulmonary Medicine, Zunyi Hospital of Traditional Chinese Medicine, Zunyi, Guizhou, China

**Keywords:** primary pulmonary lymphoma, diffuse large B-cell lymphoma, organizing pneumonia, pulmonary neoplasm, corticosteroid therapy, bronchoscopy, immunohistochemistry, extranodal lymphoma

## Abstract

Primary pulmonary lymphoma represents an uncommon extranodal manifestation of non-Hodgkin lymphoma, with atypical clinical and radiographic features frequently leading to diagnostic challenges. Herein, we present a rare case of a 56 years-old female who presented with recurrent pyrexia. Initial thoracic computed tomography (CT) demonstrated a mass-like consolidation in the left lower lobe with bilateral pulmonary nodules. Transbronchial biopsy established organizing pneumonia; however, underlying malignancy could not be excluded. Following high-dose prednisolone therapy, clinical improvement was observed with corresponding radiographic resolution. Upon corticosteroid discontinuation, febrile recurrence developed with CT demonstrating bilateral pulmonary lesion progression. Repeat bronchoscopy revealed endobronchial lesions, though biopsy demonstrated only atypical cellular features without definitive diagnosis. Six months after initial presentation, cervical lymphadenopathy developed, and excisional lymph node biopsy confirmed diffuse large B-cell lymphoma. Following four cycles of standard R-CHOP chemotherapy with RECIST criteria assessment, partial radiographic response of pulmonary lesions was documented. This case represents a documented coexistence of primary pulmonary DLBCL and organizing pneumonia, offering unique insights into diagnostic challenges and therapeutic implications.

## 1 Introduction

Pulmonary lymphoproliferative disorders can be categorized into primary pulmonary lymphoma (PPL) or secondary pulmonary involvement, the latter resulting from either contiguous spread from adjacent lymphatic structures (mediastinum, hilar lymph nodes, or thymus) or hematogenous dissemination from extrapulmonary sites (1). PPL represents a rare variant of non-Hodgkin lymphoma (NHL) characterized by parenchymal lung involvement with or without mediastinal or hilar lymphadenopathy (2). This entity constitutes merely 0.5%–1% of primary pulmonary malignancies and 3%–4% of extranodal lymphomas, representing less than 1% of all NHL cases (3, 4). Primary diffuse large B-cell lymphoma (DLBCL) of the lung ranks as the second most prevalent PPL subtype, accounting for 10%–20% of cases, with a predominant occurrence in immunocompromised individuals (5). The diagnostic process is frequently challenging due to the non-specific nature of clinical manifestations, with definitive diagnosis typically requiring thoracotomy or computed tomography (CT)-guided percutaneous biopsy specimens (6).

Organizing pneumonia (OP) represents a distinct interstitial pneumonia subtype that encompasses both cryptogenic and secondary etiologies (7). This clinicopathological entity is characterized by the presence of granulation tissue and fibrotic plugs within small airways, alveolar ducts, and alveolar spaces (8). Radiologically, OP commonly manifests as ground-glass opacities or consolidations with peribronchovascular distribution (9). CT imaging typically reveals bilateral, migratory, patchy alveolar opacities that may demonstrate spontaneous resolution (10). Given that patients frequently present with non-specific respiratory symptoms indistinguishable from infectious pneumonia—including dyspnea, cough, and fever—definitive diagnosis necessitates histopathological confirmation through invasive procedures. The pathological hallmarks include granulation tissue buds evolving from fibrinous exudates to collagenous structures containing fibroblasts and myofibroblasts, admixed with loose connective tissue within distal airspaces (11). OP has well-established associations with various underlying conditions, including connective tissue disorders, infectious processes, post-transplantation reactions, and malignancies (12). Notably, the literature contains no documented cases of OP occurring concomitantly with primary pulmonary DLBCL. Herein, we present an exceptionally rare case of primary pulmonary DLBCL with OP as the initial clinical manifestation.

## 2 Case presentation

A 56 years-old female agricultural worker presented with pyrexia. Medical history revealed herpes zoster 1 month prior, with negative findings for respiratory manifestations, tobacco or alcohol consumption, familial oncological history, and recent travel. Serological testing for hepatitis B and C virus was negative. Thoracic CT demonstrated soft tissue masses in the left inferior and right middle pulmonary lobes with localized bronchial wall thickening, suggesting neoplastic disease with metastatic potential. Bilateral pulmonary micronodules and mild mediastinal lymphadenopathy were noted. The patient had previously declined positron emission tomography due to financial constraints. Comprehensive diagnostic imaging, including cranial magnetic resonance imaging, whole-abdominal imaging, adrenal CT, and whole-body bone scintigraphy, revealed no significant abnormalities. Bronchoscopic evaluation with endobronchial ultrasound-guided sampling of lymph nodes at stations 7, 4R, and 11R demonstrated no tracheobronchial neoplasia. Pulmonary tissue specimens exhibited focal fibrous hyperplasia, while lymph node biopsies were negative for malignancy. The patient declined further recommended pulmonary sampling and was discharged after symptomatic management. One month later, the patient presented with recurrent productive cough. Physical examination revealed no peripheral lymphadenopathy. Repeated thoracic CT showed stable findings without cervical lymphadenopathy on ultrasonography. Bronchoscopy with ultrasound-guided sampling confirmed absence of endobronchial lesions. Histopathology demonstrated alveolar architecture with interstitial fibrosis, sclerosis, lymphocytic infiltration, and alveolar macrophages—findings consistent with OP. Given multifocal pulmonary lesions and persistent oncological concerns, additional biopsies were performed. The right lung specimen supported the OP diagnosis, while the left lung sample revealed coagulative necrotic tissue suggestive of tumor necrosis. The patient declined further invasive assessment and began oral prednisolone acetate (5 mg/kg daily). Follow-up after 1 month showed significant radiographic and symptomatic improvement. Despite recommendations for continued therapy and surveillance, the patient discontinued corticosteroids after 2 months. Five months later, fever recurred with CT showing lesional progression, bilateral pulmonary cavitation, and progressive mediastinal lymphadenopathy. A second pulmonary biopsy revealed atypical cells, but bronchoscopic sampling yielded only necrotic material. One week subsequently, palpable cervical lymphadenopathy developed. Excisional lymph node biopsy established diffuse large B-cell non-Hodgkin lymphoma. Immunohistochemistry demonstrated positivity for CD20, CD79a, Ki-67 (60%), CD3, Bcl-2, focal Bcl-6, and partial MUM-1, with negativity for CD30, CD15, CD10, and CD56, and focal CD21-positive follicular dendritic cells. The patient declined bone marrow assessment. Comprehensive evaluation confirmed primary pulmonary diffuse large B-cell lymphoma of non-germinal center phenotype, stage IIE. The patient received standard R-CHOP chemotherapy with partial response on post-treatment evaluation. The timeline of key clinical events is summarized in Table 1.

**TABLE 1:** Timeline of key clinical events.

Time point	Clinical event
Day 0	Initial presentation with pyrexia
Day 1	First thoracic CT showing soft tissue masses in left lower and right middle lobes
Day 3	First bronchoscopic examination with no significant endobronchial lesions
Day 5	Patient declined PET-CT due to financial constraints
Day 30	Patient returned with recurrent productive cough
Day 32	Repeat thoracic CT showing stable findings
Day 35	Second bronchoscopy confirming organizing pneumonia
Day 37	Lung biopsy showing coagulative necrotic tissue in left lung
Day 38	Initiation of oral prednisolone (5 mg/kg/day)
Day 68	Follow-up showing significant clinical and radiographic improvement
Day 98	Patient self-discontinued corticosteroid therapy
Day 248	Recurrent fever with CT showing disease progression and cavitation
Day 250	Third bronchoscopy showing bronchial stenosis and neoplastic infiltration
Day 257	Development of palpable cervical lymphadenopathy
Day 259	Lymph node biopsy confirming diffuse large B-cell lymphoma
Day 260	Initiation of R-CHOP chemotherapy
Day 380	Partial response documented after 4 cycles of chemotherapy

Patient perspective: the patient reported significant emotional distress during the prolonged diagnostic journey, expressing frustration with the initial misdiagnosis and disappointment in having to discontinue treatment due to financial constraints. The patient described severe disruption to her daily agricultural work and family responsibilities, particularly during periods of fever and respiratory symptoms. Following lymphoma diagnosis, the patient expressed relief at having a definitive diagnosis but anxiety about treatment outcomes. After experiencing symptom improvement with chemotherapy, the patient reported renewed hope and emphasized the importance of thorough diagnostic evaluations despite initial negative findings.

## 3 Discussion

The pulmonary parenchyma represents a frequent site of metastatic involvement in lymphoproliferative malignancies, with documented prevalence reaching 38% in HL and 24% in NHL (13). Conversely, PPL is rare and defined by lymphoma cells primarily in lung tissue with little or no hilar lymph node involvement at diagnosis or within 3 months (14). Within the spectrum of primary pulmonary NHL, DLBCL comprises approximately 10% of cases, demonstrating predilection for immunocompromised hosts (15). In the present case, despite documented human immunodeficiency virus seronegativity, the patient’s recent herpes zoster infection suggests underlying immunological perturbation.

The exact cause of primary pulmonary DLBCL is still unclear, though several theories exist. A predominant hypothesis suggests that chronic inflammatory pulmonary conditions precipitate aberrant clonal lymphoid proliferation within the pulmonary microenvironment (16). The clinical manifestations of both interstitial pneumopathies and pulmonary neoplastic processes demonstrate substantial overlap and lack pathognomonic features; approximately one-third of patients remain clinically asymptomatic. Symptomatic presentations typically encompass non-specific respiratory manifestations including cough, pyrexia, dyspnea, and thoracic discomfort (17). Definitive differentiation between these pathological entities necessitates histopathological assessment rather than clinical parameters alone. The initial pulmonary parenchymal biopsy in this patient established OP, a condition with recognized secondary etiologies. While associations between OP and HL have been documented in the literature (18), analogous correlations with NHL remain undescribed. Glucocorticoid therapy constitutes the cornerstone of OP management but may confound lymphoma diagnosis while simultaneously exerting cytoreductive effects on lymphomatous tissue (19), potentially obscuring the temporal progression and delaying definitive diagnosis in cases of coexistent pathologies.

**FIGURE 1:**
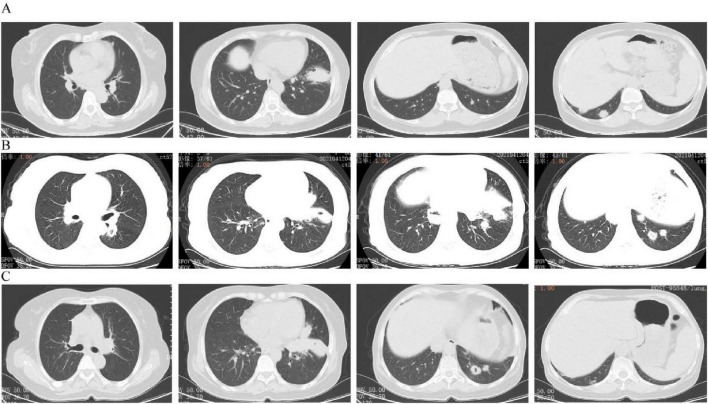
Chest imaging progression. **(A)** Initial computed tomography (CT) scan showing masses in left lower and right middle lobes with bronchial thickening, suggesting possible cancer. Multiple small nodules visible in both lungs. **(B)** CT scan 5 months after stopping steroids showing significant worsening with larger lesions, multiple cavities, and enlarged lymph nodes. **(C)** CT scan after 4 cycles of R-CHOP showing shrinkage of lung lesions and partial resolution of cavities, indicating good response to treatment.

Primary pulmonary DLBCL exhibits heterogeneous radiographic manifestations, including nodular lesions, mass-like consolidations, and hilar or mediastinal lymphadenopathy (20). Conventional radiography and computed tomography typically demonstrate bronchial involvement with variable lobar atelectasis (21), as seen in Figure 1A. Cavitation and necrosis represent characteristic features of primary DLBCL (22), as demonstrated in Figure 1B. In this case, the development of multiple pulmonary cavities with irregular, nodular internal margins during disease progression likely reflects necrotic transformation of tumor tissue with endobronchial evacuation, with significant improvement observed after treatment (Figure 1C). The subsequent bronchoscopic identification of endobronchial neoplastic infiltration correlates with these radiographic findings.

**FIGURE 2:**
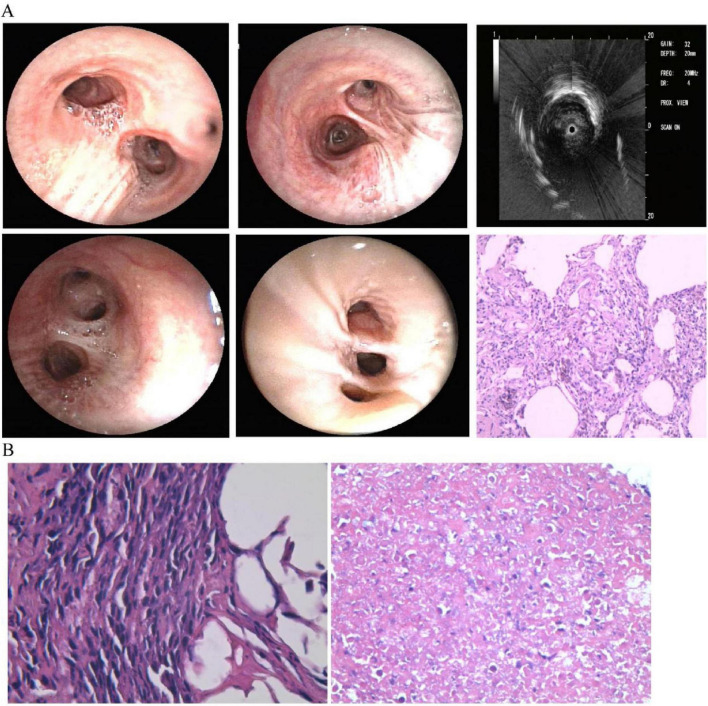
First bronchoscopy and lung biopsy findings. **(A)** Normal-appearing airways with ultrasound showing abnormalities in left lower lobe. Biopsy shows tissue scarring with lymphocyte infiltration and macrophages in air spaces, typical of organizing pneumonia. **(B)** Right lung biopsy (left panel) shows inflammatory changes; left lung biopsy (right panel) shows extensive tissue death suggestive of tumor necrosis.

**FIGURE 3:**
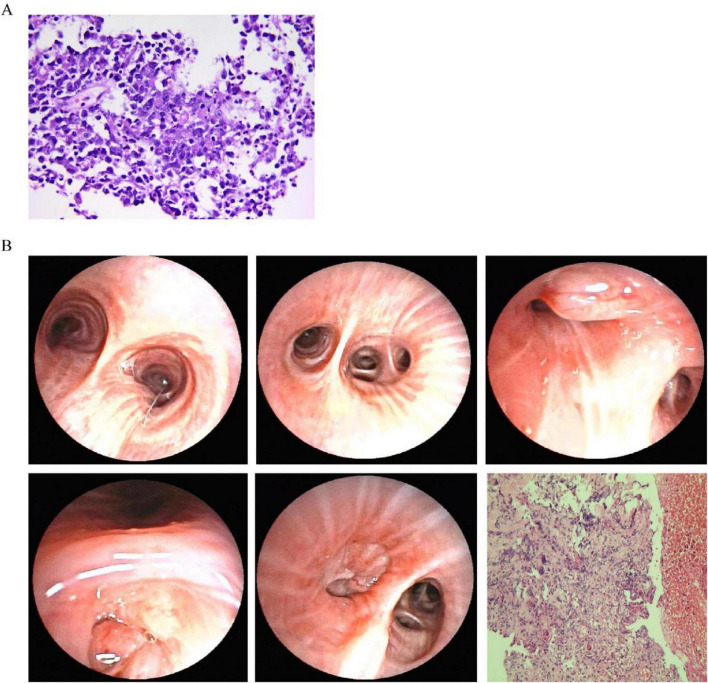
Second lung biopsy and bronchoscopy findings. **(A)** Second right lower lobe biopsy shows inflammatory tissue with abnormal small round cells having irregular nuclei and occasional cell division. Further testing needed but limited by small sample size. **(B)** Second bronchoscopy shows narrowing of left bronchial branches with tumor-like growth in multiple segments. Biopsies show dead tissue, inflammation, and some preserved cartilage and lung tissue.

When thoracic imaging demonstrates mass-like parenchymal abnormalities, tissue acquisition becomes imperative for definitive diagnosis. The diagnosis of pulmonary lymphoma is often hampered by technical challenges that frequently delay accurate classification. Bronchoscopic approaches with bronchoalveolar lavage (BAL) and transbronchial lung biopsy (TBLB) may provide specimens (23), as shown in Figure 2A, though with limited diagnostic yield—notably, 42% of patients with eventual lymphoma diagnosis demonstrate initially non-diagnostic or inflammatory findings on bronchoscopic evaluation (24). These limitations stem primarily from insufficient tissue volume and the presence of necrotic specimens, particularly challenging in DLBCL where extensive necrosis is characteristic (25). In our case, BAL cytological analysis revealed no malignant cells, while TBLB demonstrated only interstitial fibrosis and lymphocytic infiltration consistent with OP (Figure 2B). Subsequent CT-guided percutaneous lung biopsy demonstrated coagulative necrosis and lymphocytic infiltration raising suspicion for lymphomatous involvement, but limited specimen quantity precluded comprehensive immunophenotypic analysis (26). Studies show that definitive diagnosis typically follows a median diagnostic delay of 3 months (range 5–36 months), often requiring multiple sampling attempts until a large tissue specimen is obtained, as evidenced in our case with repeated bronchoscopic examinations (Figure 3). While less invasive methods like transbronchial lung cryobiopsy (TBLC) combined with endobronchial ultrasound and guide sheath can provide larger samples and reduce the need for surgery, these approaches may remain insufficient in complex cases. Strategies to overcome these limitations include CT-guided core needle biopsy with larger-gauge needles (18–19G), sampling from multiple regions to account for tumor heterogeneity, and ancillary studies including immunohistochemistry, flow cytometry, cytogenetics, and molecular analysis for B-cell clonality (25, 27). When these approaches prove insufficient, surgical lung biopsy via video-assisted thoracoscopic surgery offers superior diagnostic yield (> 90%) though with increased procedural risk. In our patient, while surgical lung biopsy might have provided optimal diagnostic material, the development of cervical lymphadenopathy 6 months after initial presentation facilitated definitive diagnosis through less invasive excisional lymph node biopsy. This case highlights two critical clinical lessons: first, clinicians must maintain vigilance for underlying malignancy in steroid-unresponsive or relapsing organizing pneumonia; second, when pulmonary samples yield inconclusive results, lymph node biopsy—even at distant sites—can provide definitive diagnosis while avoiding the morbidity of surgical lung procedures. Multidisciplinary discussion before diagnostic attempts is recommended to optimize sampling strategies and minimize unnecessary invasive procedures.

**FIGURE 4:**
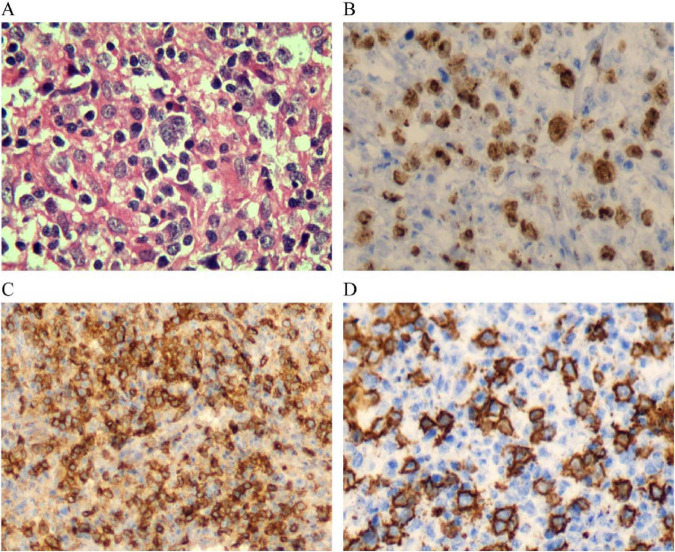
Lymph node biopsy confirming lymphoma. **(A)** Lymph node biopsy shows cancerous cells in lymphatic tissue. **(B–D)** Special staining shows positive results for Ki-67, CD79a, and CD20, confirming diagnosis of diffuse large B-cell lymphoma.

Primary pulmonary DLBCL exhibits diverse histopathological characteristics; Ki-67 proliferation index, a quantitative measure of cellular proliferation, frequently demonstrates elevated expression levels ranging from 80% to 90%, indicating substantial proliferative activity (Figure 4A). This heightened proliferative capacity correlates with the observed clinical course in this patient, wherein discontinuation of corticosteroid therapy precipitated dramatic radiographic progression. Immunohistochemical positivity for CD79a suggests B-lymphocyte lineage derivation (Figure 4B). The cardinal immunophenotypic characteristic of DLBCL encompasses expression of B-lineage markers, predominantly CD20 and PAX5, with variable morphological heterogeneity (Figures 4C, D). The patient’s immunohistochemical profile demonstrated strong CD20 immunoreactivity, consistent with established diagnostic criteria (28).

In the present case, definitive diagnosis was established 6 months after initial symptom manifestation. This diagnostic delay can be attributed to multiple factors, including the inherent rarity of primary pulmonary lymphoma with consequent limited clinical recognition, socioeconomic constraints precluding comprehensive diagnostic evaluation, and deferral of invasive diagnostic procedures. Additionally, the non-specific clinical manifestations, heterogeneous radiographic appearances, and suboptimal diagnostic yield of conventional needle biopsy techniques for lymphoproliferative disorders contributed to diagnostic delay. Notably, primary pulmonary DLBCL typically demonstrates excellent therapeutic response to systemic chemotherapy, with greater than 90% of patients achieving complete response following conventional chemotherapeutic regimens (29). In this case, institution of systemic chemotherapy resulted in significant clinical improvement, with partial response documented on therapeutic reassessment.

In conclusion, primary pulmonary DLBCL represents an uncommon variant of extranodal NHL characterized by non-specific clinical and radiographic manifestations. To our knowledge, concomitant presentation with organizing pneumonia has not been previously documented in the literature. We present this unusual case to enhance clinicopathological recognition and facilitate diagnostic and therapeutic optimization for patients with primary pulmonary neoplastic processes. Key clinical lessons include maintaining vigilance for occult malignancy in steroid-unresponsive organizing pneumonia cases and recognizing the critical role of lymph node biopsy in unresolved diagnostic scenarios.

## Data Availability

The original contributions presented in this study are included in this article/supplementary material, further inquiries can be directed to the corresponding author.
